# Serum adenosine deaminase levels are associated with diabetic kidney disease in type 2 diabetic patients

**DOI:** 10.1530/EC-21-0306

**Published:** 2021-07-28

**Authors:** Chun-feng Lu, Wang-shu Liu, Xiao-qin Ge, Feng Xu

**Affiliations:** 1 Department of Endocrinology, Affiliated Hospital 2 of Nantong University and First People’s Hospital of Nantong City, Nantong, China

**Keywords:** type 2 diabetes, adenosine deaminase, diabetic kidney disease, urinary albumin-to-creatinine ratio, estimated glomerular filtration rate

## Abstract

The aim of the present study was to evaluate the association between adenosine deaminase (ADA) levels and diabetic kidney disease (DKD) in patients with type 2 diabetes (T2D). In this study, patients with T2D who had been screened for DKD were recruited. Patients with an estimated glomerular filtration rate (eGFR) < 60 mL/min/1.73 m^2^ or a urinary albumin-to-creatinine ratio (UACR) ≥ 30 mg/g for 3 months were identified as having DKD. The prevalence of DKD was 13.3%, and the range of serum ADA levels was 4–37 U/L. Serum ADA levels were positively associated with cystatin C levels and UACR (*r = *0.295 and *r = *0.302, respectively, both *P <* 0.05) and negatively associated with eGFR (*r = *−0.342, *P <* 0.05). The proportion of participants with DKD increased significantly from 3.8% in the first tertile (T1) to 13.6% in the second tertile (T2) and 25.9% in the third tertile (T3) of ADA (*P* for trend < 0.001). After adjusting for clinical risk factors for DKD via multiple logistic regression, the corresponding odds ratios (ORs) of DKD for the participants in T2 and T3 vs those in T1 of ADA were 5.123 (1.282–20.474) and 10.098 (1.660–61.431), respectively. Receiver operating characteristic (ROC) analysis revealed that the optimal cutoff value of ADA to indicate DKD was 10 U/L. Its corresponding sensitivity and specificity were 75.5 and 56.4%, respectively. Our results demonstrated that serum ADA levels were closely associated with DKD and partly reflect the risk of DKD in patients with T2D.

## Introduction

Diabetes has become a major public health problem in China, and a study conducted in 2013 showed that the prevalence of diabetes in China was as high as 10.9% (1). Type 2 diabetes (T2D) accounts for more than 95% of diabetic cases in China (2). Diabetic kidney disease (DKD), one of the most common diabetic microvascular complications, is a major cause of the end-stage renal disease (ESRD) and is associated with increased mortality in diabetic patients (3). As the prevalence of T2D has risen, the prevalence of DKD has also increased. A meta-analysis of observational studies shows that the prevalence of DKD in Chinese patients with T2D is 21.8% (4). Even worse, the current management for DKD can only slightly delay the progression of DKD but does not reduce the proportion of DKD patients who progress to ESRD (5). In addition, albuminuria is the most reliable early diagnostic biomarker of DKD, but mounting evidence reveals that in diabetes, albuminuria does not always precede renal function decline (6). A diagnosis of DKD depending on albuminuria may delay the diagnosis and treatment of some patients with DKD. Therefore, there is an urgent need to seek new therapeutic targets and diagnostic markers for DKD.

Adenosine deaminase (ADA), a polymorphic enzyme, is expressed in all human tissues and plays an important role in regulating adenosine concentration by catalyzing the irreversible deamination of adenosine to inosine (7). Adenosine is an important paracrine inhibitor of inflammation (8). Under physiological conditions, the concentration of adenosine is relatively low, but when cells are subjected to stress, the concentration of adenosine increases to more than 100 times the baseline concentration (9). An increased adenosine concentration can play an anti-inflammatory and cell-protective role by inhibiting the activation of macrophages and the production of cytokines and chemokines (10). Thus, ADA may contribute to inflammation by reducing the extracellular adenosine concentration. Correspondingly, serum ADA levels were significantly increased in some inflammatory diseases, such as acute lymphoblastic leukemia, autoimmune hepatitis, inflammatory bowel disease, and rheumatic disease (11). Recently, growing evidence has shown that inflammation plays a key role in the onset and progression of DKD (12). Accordingly, we speculated that serum ADA levels are a potential risk factor for DKD. Although multiple studies have shown that serum ADA levels are significantly increased in patients with diabetes (13, 14, 15), no study has reported the relationship between serum ADA levels and DKD in patients with T2D.

Therefore, the aim of the present study was to estimate whether serum ADA levels were related to DKD in type 2 diabetic patients.

## Methods

### Study design and participants

This cross-sectional study enrolled patients with T2D at the inpatient department of the Second Afﬁliated Hospital of Nantong University between July 2020 and December 2020. The inclusion criterion was T2D diagnosed based on the statement of the American Diabetes Association in 2011 (16). The exclusion criteria included the following: (1) type 1 diabetes (T1D), (2) previous use of drugs that affect glycemic metabolism, that is, steroids, (3) previous and current malignant tumors, (4) chronic hepatitis and heart failure, (5) acute diabetic complications, that is, diabetic ketoacidosis and (6) other kidney diseases and urinary tract infection. The study completely complied with the Declaration of Helsinki, and all subjects provided written informed consent. The study was approved by the medical research ethics committee of the Second Afﬁliated Hospital of Nantong University. Finally, 400 patients with T2D were enrolled in the present study.

### Basic data collection

Upon enrollment, information on age, sex, medical history and anthropometry parameters was obtained for all participants through interviews and examinations by experienced physicians. BMI was calculated as the weight (kg)/height (m) squared. After each participant rested for at least 30 min, blood pressure was measured by a standard mercury sphygmomanometer, and the average of three recordings was recorded.

### Laboratory examination

The next morning after enrollment, fasting blood samples were collected to measure laboratory parameters, and fresh morning first-void urine samples were collected to measure urinary albumin and creatinine level. Serum ADA, total protein, albumin, globulin, triglyceride (TG), total cholesterol (TC), low-density lipoprotein cholesterol (LDL-c), high-density lipoprotein cholesterol (HDL-c), blood urea nitrogen (BUN), creatinine (Cr), cystatin C and uric acid (UA) levels were measured with an automated biochemical analyzer (Model 7600, Hitachi). HbA1c levels were measured with an ion exchange-based HPLC method in a hemoglobin analysis system (D-10, Bio-Rad). The urinary albumin/creatinine ratio (UACR) was calculated according to the equation of urinary albumin level/urinary creatinine level. eGFR was calculated based on the Chronic Kidney Disease Epidemiology Collaboration (CKD-EPI) creatinine–cystatin C equation (2012) (17).

### Diagnostic criteria of DKD

According to the American Diabetes Association Consensus in 2014, DKD was defined as an eGFR < 60 mL/min/1.73 m^2^ or a UACR ≥ 30 mg/g for more than 3 months (18). Patients with a UACR ≥ 30 mg/g upon enrollment were reexamined for UACR 3 months later and were diagnosed with DKD if the UACR was still higher than 30 mg/g.

### Statistical analyses

Clinical variables are shown for all type 2 diabetic subjects and for the tertiles of serum ADA levels. Continuous variables with normal and skewed distributions and categorical variables were described as the mean ± s.d., median (25 and 75% interquartile), and frequency (percentage), respectively. One-way ANOVA, the Kruskal–Wallis test, and the chi-square test were used to compare differences in normally distributed data, skewed data and categorical data among the three subgroups based on ADA tertiles. The correlations of ADA levels with clinical parameters were analyzed by Spearman’s bivariate correlation analysis. Multivariate logistic regression analysis models were applied to evaluate the independent impact of ADA on the risk of DKD, and the corresponding odds ratios (ORs) and 95% CIs were provided. Furthermore, receiver operating characteristic (ROC) analysis was conducted to analyze the ability of ADA levels to indicate the presence of DKD, and the corresponding cutoff value was provided. Data analyses were performed using SPSS statistical software 18.0 (IBM SPSS Inc.). A value of *P* < 0.05 was considered to be statistically significant.

## Results

### Basic characteristics

The clinical characteristics of the participants are presented in Table 1. Among the 400 recruited patients with T2D, the prevalence of DKD was 13.3%. The range of ADA levels in all participants was 4–37 U/L. From the first to third tertile, age, diabetes duration, systolic blood pressure (SBP), HbA1c level, total protein concentration, globulin level, UA level, cystatin C level, UACR, percentage of DKD, erythrocyte sedimentation rate (ESR), C-reactive protein (CRP) level, fibrinogen (Fg) level, and D-dimer level significantly increased (*P* for trend < 0.05), whereas the percentage of females, percentage of patients using insulin, and eGFR decreased (*P* for trend > 0.05). There were significant differences in the use of metformin, acarbose, insulin sensitizers and statins among the tertiles of ADA levels (*P* for trend < 0.05). However, BMI, diastolic blood pressure (DBP), use of insulin secretagogues and dipeptidyl peptidase 4 (DPP-4) inhibitors, antihypertensive treatments, lipid profile, BUN level, and Cr level did not show any difference among the tertiles of ADA levels (*P* for trend > 0.05).

**Table 1:** Clinical characteristics of the study participants.

Variables	Total	T1	T2	T3	*P* for trend
ADA (U/L)	10 (8–13)	<10	10–12	>12	
*n*	400	156	132	112	
Age (years)	56.77 ± 12.92	52.42 ± 11.26	58.17 ± 13.29	61.16 ± 12.88	<0.001
Male, *n* (%)	249 (62.3)	114 (73.1)	80 (60.6)	55 (49.1)	<0.001
Diabetes duration (years)	5 (1–10)	3 (1–9)	7 (1–10)	7 (1–18)	0.001
BMI (kg/m^2^)	25.78 ± 4.12	25.36 ± 3.73	25.78 ± 3.66	26.42 ± 5.07	0.227
SBP (mmHg)	132.0 (122.3–145.0)	130.0 (120.0–140.0)	131.5 (122–144.8)	135.5 (126.0–153.0)	0.003
DBP (mmHg)	80.49 ± 10.50	80.96 ± 10.79	79.87 ± 9.71	80.57 ± 11.03	0.678
Antidiabetic treatment					
Insulin treatment, *n* (%)	249 (62.3)	114 (73.1)	80 (60.6)	55 (49.1)	<0.001
Metformin, *n* (%)	161 (40.3)	69 (44.2)	60 (45.5)	32 (28.6)	0.012
Acarbose, *n* (%)	36 (9.0)	18 (11.5)	15 (11.4)	3 (2.7)	0.022
Insulin secretagogues, *n* (%)	123 (30.8)	49 (31.4)	43 (32.6)	31 (27.7)	0.693
Insulin sensitisers, *n* (%)	28 (7.0)	10 (6.4)	15 (11.4)	3 (2.7)	0.028
DPP-4 inhibitors, *n* (%)	16 (4.0)	7 (4.5)	3 (2.3)	6 (5.4)	0.436
Antihypertensive treatments					
CCB, *n* (%)	91 (22.8)	30 (19.4)	32 (24.2)	29 (25.9)	0.405
ARB, *n* (%)	74 (18.5)	23 (14.7)	29 (22.0)	22 (19.6)	0.271
β-blockers, *n* (%)	20 (5.0)	6 (3.8)	5 (3.8)	9 (8.0)	0.221
Diuretics, *n* (%)	25 (6.3)	9 (5.8)	5 (3.8)	11 (9.8)	0.145
Statin medications, *n* (%)	15 (3.8)	2 (1.3)	10 (7.6)	3 (2.7)	0.015
HbA1c (%)	9.28 ± 2.17	8.70 ± 1.98	9.49 ± 2.15	9.86 ± 2.27	<0.001
Total protein (g/L)	62.75 ± 6.18	61.39 ± 4.93	63.30 ± 5.83	64.00 ± 7.67	0.001
Albumin (g/L)	38.83 ± 3.99	38.91 ± 4.05	39.19 ± 3.74	38.29 ± 4.15	0.203
Globulin (g/L)	23.6 (21.0–26.2)	22.3 (20.1–24.1)	23.6 (20.9–26.3)	25.5 (23.6–29.2)	<0.001
TG (mmol/L)	1.54 (1.04–2.58)	1.62 (1.09–2.57)	1.47 (0.93–2.46)	1.61 (1.09–3.14)	0.360
TC (mmol/L)	4.51 ± 1.19	4.54 ± 1.07	4.47 ± 1.10	4.52 ± 1.44	0.903
HDL-c (mmol/L)	1.14 (0.98–1.35)	1.14 (1.02–1.31)	1.15 (1.00–1.41)	1.14 (0.93–1.37)	0.399
LDL-c (mmol/L)	2.77 ± 0.90	2.83 ± 0.88	2.77 ± 0.92	2.70 ± 0.89	0.532
BUN (mmol/L)	5.23 (4.24–6.40)	5.13 (4.14–6.35)	5.34 (4.43–6.26)	5.25 (4.24–6.99)	0.451
Cr (umol/L)	56.0 (49.0–66.0)	55.0 (50.0–63.0)	57.0 (50.3–66.0)	55.5 (48.0–74.8)	0.271
UA (umol/L)	308.18 ± 95.76	298.37 ± 85.74	301.92 ± 87.99	329.21 ± 113.71	0.022
Cystatin C (mg/L)	0.74 (0.60–0.91)	0.69 (0.55–0.79)	0.78 (0.64–0.94)	0.84 (0.67–1.10)	<0.001
UACR (mg/g)	13.9 (7.5–46.9)	9.3 (5.5–25.8)	16.0 (9.0–57.3)	19.1 (9.1–116.3)	<0.001
eGFR (mL/min/1.73 m^2^)	108.10 ± 28.02	119.01 ± 22.17	105.77 ± 24.44	95.66 ± 31.34	<0.001
DKD, *n* (%)	53 (13.3)	6 (3.8)	18 (13.6)	29 (25.9)	<0.001
ESR (mm)	6 (3–11)	5 (2–8)	6 (3–12)	10 (6–20)	<0.001
CRP (mg/L)	0.39 (0.06–2.41)	0.16 (0.04–0.77)	0.46 (0.07–3.20)	1.22 (0.19–5.24)	<0.001
Fg (g/L)	2.42 (2.06–2.97)	2.32 (1.98–2.63)	2.38 (2.11–2.97)	2.64 (2.14–3.31)	<0.001
D-dimer (ug/L)	220 (190–390)	190 (190–320)	225 (190–378)	275 (190–573)	0.002

Normally distributed values in the table are given as the mean ± s.d., skewed distributed values are given as the median (25 and 75% interquartiles), and categorical variables are given as frequency (percentage). ANOVA, the Kruskal–Wallis test and the Chi squared test were conducted to determine *P* values for normally distributed continuous variables, skewed continuous variables and categorical variables, respectively.

ACEI, angiotensin-converting enzyme inhibitors; ADA, adenosine deaminase; ARB, angiotensin receptor blockers; BMI, body mass index; BUN, blood urea nitrogen; CCB, calcium channel blockers; Cr, creatinine; CRP, C-reactive protein; DKD, diabetic kidney disease; DPP-4, inhibitors dipeptidyl peptidase-4 inhibitors; eGFR, estimated glomerular filtration rate; ESR, erythrocyte sedimentation rate; Fg, fibrinogen; HbA1c, glycosylated hemoglobin A1c; HDL-c, high density lipoprotein cholesterol; LDL-c, low density lipoprotein cholesterol; SBP/DBP, systolic/diastolic blood pressure; TC, total cholesterol; TG, triglyceride; UA, uric acid.

### Relationship between ADA and clinical parameters

As illustrated in the Table 2, serum ADA levels were positively associated with age, diabetes duration, SBP, insulin treatment, HbA1c level, total protein concentration, globulin concentration, UA level, cystatin C level, UACR, ESR level, CRP level, Fg level and D-dimer level (*r* = 0.198, *r* = 0.163, *r* = 0.166, *r* = 0.188, *r* = 0.266, *r* = 0.216, *r* = 0.422, *r* = 0.106, *r* = 0.295, *r* = 0.302, *r* = 0.388, *r* = 0.314, *r* = 0.250, *r* = 0.234, respectively, all *P* < 0.05) and negatively associated with metformin treatment, acarbose treatment and eGFR (*r* = −0.156, *r* = −0.112, *r* = −0.342, respectively, all *P* < 0.05). However, there were no significant correlations between ADA levels and BMI, DBP, other antidiabetic treatments, antihypertensive treatments, statin medication use, lipid profile, Cr level or BUN level (all *P >* 0.05).

**Table 2:** Relationships between ADA and clinical parameters.

Variables	*r*	*P-*value
Age	0.198	<0.001
Diabetes duration	0.163	0.001
BMI	0.085	0.157
SBP	0.166	0.001
DBP	0.005	0.919
Insulin treatment	0.188	<0.001
Metformin	−0.156	0.002
Acarbose	−0.112	0.025
Insulin secretagogues	−0.076	0.132
Insulin sensitisers	−0.071	0.154
DPP-4 inhibitors	−0.006	0.898
CCB	0.055	0.275
ARB	0.076	0.128
ACEI	−0.027	0.589
β-blockers	0.060	0.231
Diuretics	0.066	0.191
Statins medications	0.042	0.401
HbA1c	0.266	<0.001
Total protein	0.216	<0.001
Globulin	0.422	<0.001
TG	0.042	0.401
TC	0.022	0.663
HDL-c	−0.034	0.501
LDL-c	−0.051	0.313
BUN	0.039	0.441
Cr	0.072	0.148
UA	0.106	0.033
Cystatin C	0.295	<0.001
eGFR	−0.342	<0.001
UACR	0.302	<0.001
ESR	0.388	<0.001
CRP	0.314	<0.001
Fg	0.250	<0.001
D-dimer	0.234	<0.001

*r* spearman’s correlation coefficient.

### Proportion and ORs of DKD according to ADA tertiles

The proportion of participants with DKD increased significantly from 3.8% in T1 to 13.6% in T2 and 25.9% in T3 of ADA (*P* for trend < 0.001). Table 3 also shows the ORs of DKD according to the ADA tertiles. Compared with the OR of DKD for the participants in T1 of ADA, the ORs for the participants in T2 and T3 of ADA were 3.947 (95% CI 1.518–10.263) and 8.735 (3.484–21.897), respectively. After adjusting for other clinical risk factors for DKD via multiple logistic regression, the corresponding ORs of DKD for the participants in T2 and T3 vs those in T1 of ADA were 5.123 (1.282–20.474) and 10.098 (1.660–61.431), respectively.

**Table 3:** Proportion and odds ratios (ORs) of DKD according to ADA tertiles (95% CI).

	ADA tertiles			*P* for trend
	T1 (<10 U/L)	T2 (10–12 U/L)	T3 (>12 U/L)	
n	156	132	112	
DKD, *n* (%)	6 (3.8)	18 (13.6)	29 (25.9)	<0.001
Model 0	1-reference	3.947 (1.518–10.263)	8.735 (3.484–21.897)	<0.001
Model 1	1-reference	2.624 (0.899–7.655)	4.894 (1.627–14.722)	0.009
Model 2	1-reference	3.217 (1.025–10.093)	5.604 (1.652–19.009)	0.005
Model 3	1-reference	5.123 (1.282–20.474)	10.098 (1.660–61.431)	0.004

Model 0: unadjusted model; Model 1: adjusted for age, male, diabetic duration, BMI; Model 2: additionally adjusted for SBP, DBP, HbA1c, TG, TC, HDL-c, LDL-c; Model 3: additionally adjusted for antidiabetic treatments, antihypertensive treatments, statin medications.

### ROC analysis to explore the cutoff ADA value to diagnose DKD

**Figure 1:**
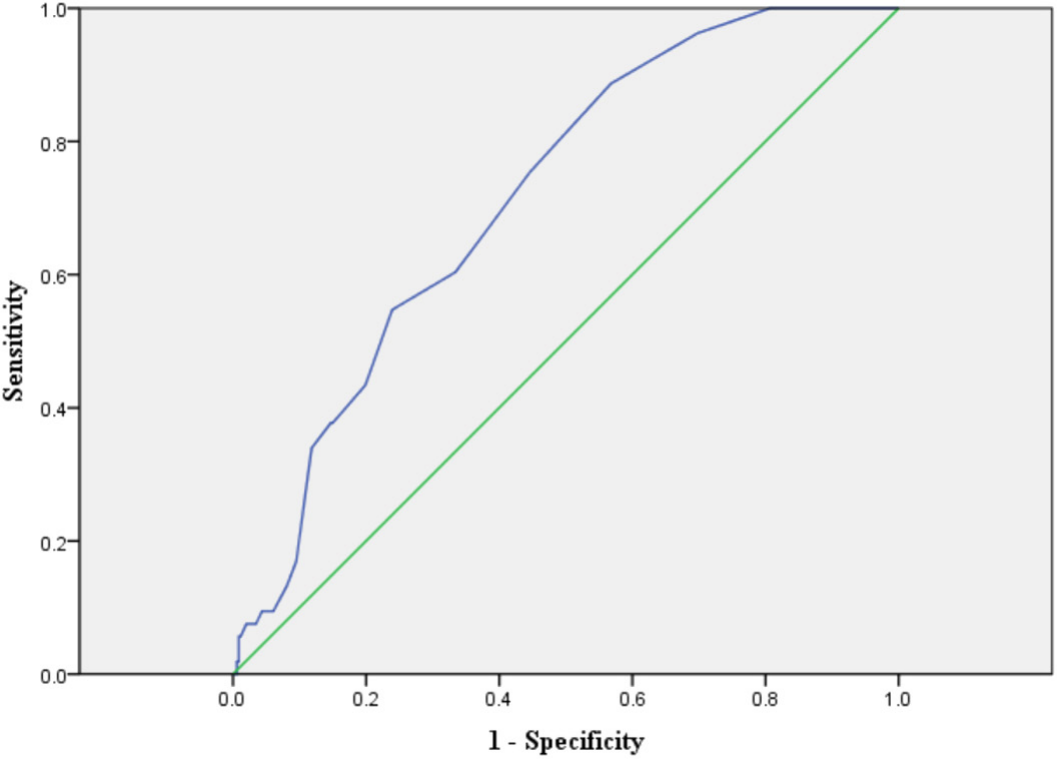
ROC analysis to analyze the ability of ADA to indicate confirmed DKD. ROC analysis to assess the ability of ADA to indicate confirmed DKD was 0.716 (95% CI 0.652–0.781). Optimal cutoff value of ADA was 10 U/L to indicate DKD; Youden index = 0.319, sensitivity = 75.5% and specifcity = 56.4%.

ROC analysis was further conducted to explore the cutoff ADA value to indicate confirmed DKD cases. The optimal cutoff value of ADA to indicate DKD was 10 U/L. The corresponding area under the curve (AUC) to indicate DKD was 0.716 (95% CI 0.652–0.781), its Youden index was 0.319, its sensitivity was 75.5%, and its specificity was 56.4% (Fig. 1).

## Discussion

In the current study, we evaluated the association of serum ADA levels and DKD in type 2 diabetic patients. The main findings of this study are as follows: first, among the recruited type 2 diabetic patients, the prevalence of DKD was 13.3% (n = 32); secondly, an increased serum ADA level was identified as a significant independent contributor to DKD; thirdly, patients in the second and third ADA tertiles were at higher risk for DKD than those in the first ADA tertile, with multiple-adjusted ORs of 5.123 (1.282–20.474) and 10.098 (1.660–61.431), respectively; fourthly, the optimal cutoff value of ADA to indicate DKD was 10 U/L, and its corresponding sensitivity and specificity were 75.5 and 56.4%, respectively. Our results revealed that a high serum ADA level was closely associated with DKD in patients with T2D.

Measurement of ADA levels is routine in the detection of clinical hepatic function, and recent studies have shown that it has a potential role in the assessment of metabolic diseases, inflammatory diseases, cardiovascular diseases and so on. Multiple studies enrolling patients with T2D have found that serum ADA levels are significantly higher in those with T2D than in those without T2D and are associated with poor glycemic control and insulin resistance (13, 14, 15). Adenosine, a substrate of ADA, can promote glucose uptake into cells, while high levels of ADA can inhibit glucose uptake into cells by degrading adenosine, thereby aggravating insulin resistance (18). Further, Gowda and his colleagues (19) found that patients with T2D who received metformin treatment might have reduced serum ADA levels through improved insulin resistance. These data are in agreement with our study, in which serum ADA levels were positively correlated with HbA1c and diabetes duration and the proportions of type 2 diabetic patients treated with metformin and insulin sensitizers were lower in the third ADA tertile than in the first and second tertiles.

Hyperglycemia interferes with nitric oxide synthesis by activating protein kinase C and triggers inflammatory responses by promoting the release of cytokines and adhesion molecules (20). Some proinflammatory cytokines, such as TNF-α, IL-1 and IL-6, can promote the expression of procoagulant molecules and inhibit the expression of anticoagulant molecules, eventually leading to hypercoagulability (21). Therefore, hyperglycemia is closely related to inflammation and hypercoagulability in patients with T2D. In the present study, we observed that serum ADA levels were significantly positively correlated with CRP levels, ESR, Fg levels and D-dimer levels. Similar to our study, Yu *et al.* (22) also found that serum ADA levels were positively correlated with CRP levels in patients with T2D. ADA is most abundantly expressed in lymphoid tissues and is indispensable for regulating the proliferation and differentiation of T lymphocytes and the maturation and function of monocytes and macrophages (23). As mentioned before, serum ADA levels are significantly elevated in some inflammatory diseases, and inhibition of ADA may contribute to reducing inflammation (24). Thus, high serum ADA levels in patients with T2D may partly reflect the degree of hyperglycemia, inflammation, and hypercoagulability.

DKD is a multifactorial disease, and disordered glucose and lipid metabolism and alterations in hemodynamics, inflammation and oxidative stress are all involved in its onset and progression (25), while serum ADA levels are closely associated with hyperglycemia, inflammation, and hypercoagulability. Based on this, we hypothesized that increased serum ADA levels may result in an increased risk for DKD in patients with T2D. In support of this hypothesis, our study found that serum ADA levels were significantly positively correlated with UACR and cystatin C levels and negatively correlated with eGFR, while UACR, cystatin C levels and eGFR were important evaluation indexes of DKD (26). Moreover, multivariate logistic regression analysis demonstrated that patients in the second and third ADA tertiles were at higher risk for DKD than those in the first ADA tertile, and ROC analysis revealed that the optimal cutoff value of ADA to indicate DKD was 10 U/L.

Several possible mechanisms to explain the link between high serum ADA levels and increased DKD risk are available. Inflammation plays a major role in the progression of DKD, and macrophages are the initiators of inflammation (27). Radica *et al.* found that the magnitude of macrophage infiltration in the kidney is closely related to the decline in renal filtration function (28). As early as 2003, William *et al.* established a rat model of fecal peritonitis and found that the expression of ADA was significantly increased in tissues rich in macrophages (29). In experimental diabetic retinopathy (DR), ADA promotes the progression of DR by participating in the destruction of the blood-retinal barrier (BRB) through macrophage-derived cytokines, and inhibition of ADA can preserve BRB function (30). Therefore, increased serum ADA in patients with T2D can accelerate the onset and progression of DKD by inducing macrophage infiltration in the kidney and the expression of macrophage-derived cytokines. In addition, adenosine, the substrate of ADA, has been shown in several studies to have renal and cardiovascular protective effects. In the kidney, adenosine can regulate the release of renin, renal vascular tension, and glomerular filtration rate (31). For cardiovascular protection, adenosine can induce coronary artery dilation, scavenge oxygen free radicals, inhibit platelet activation, and maintain cholesterol balance (32). ADA may inhibit the renal protective effects of adenosine and thus aggravate renal damage in patients with T2D. Therefore, increased serum ADA may be a potential factor associated with DKD risk in patients with T2D.

Notably, our study had some limitations. First, the present study is a cross-sectional study, which cannot conclude a cause–effect relationship between high serum ADA levels and DKD in type 2 diabetic patients. Secondly, all the subjects enrolled in this study were Chinese, which limited the generalizability of our study. Thirdly, ADA mainly acts on adenosine, but this study did not simultaneously detect adenosine levels. Fourthly, the risk factors for DKD in T2D include smoking, alcohol consumption, insulin resistance and so on. Therefore, further research should be conducted to validate the results of our study and to address the above limitations.

In summary, serum ADA is a simple and readily available indicator, and increased serum ADA levels are closely associated with DKD in type 2 diabetic patients. Serum ADA may have the potential to help clinicians identify patients with T2D who are at risk for DKD.

## Declaration of interest

The authors declare that there is no conflict of interest that could be perceived as prejudicing the impartiality of the research reported.

## Funding

The study was supported by the Medical Research Project of Health Commission of Nantong (MB2020012).
